# Internal architecture of the proximal femur: calcar femorale or Adams’ arch?

**DOI:** 10.1007/s00264-023-05764-3

**Published:** 2023-03-16

**Authors:** Jan Bartoníček, Jan Alt, Ondřej Naňka

**Affiliations:** 1 Department of Orthopedics, First Faculty of Medicine, Charles University and Military University Hospital, U Vojenské Nemocnice 1200, Prague 6, Prague, 169 02 Czech Republic; 2 Institute of Anatomy, First Faculty of Medicine, Charles University, U Nemocnice 3, Prague 2, Prague, 128 00 Czech Republic

**Keywords:** Anatomy, Structure of proximal femur, Adams’ arch, Calcar femorale, History

## Abstract

**Purpose:**

The calcar femorale (femoral calcar) is used in the English literature to designate the thickened medial cortex of the femoral neck. This term is, however, incorrect, as the calcar femorale is actually quite another structure.

**Methods:**

Searching was performed in original and historic publication.

**Results:**

The importance of the thickened medial cortex of the proximal femur in femoral neck fractures was discussed already by Robert Adams in 1834–1836. Therefore, the German surgeon C.W. Streubel, in 1847, called it Adamscher Knochenbogen (Adams’ arch). Due to misspelling, this term was gradually changed to Adambogen, and at the turn of twentieth century, it was commonly used primarily in the German literature. Then, it fell into oblivion and its “renaissance” came as late as during the 1960s, again in the German literature, in connection with operative treatment of trochanteric fractures.

**Conclusions:**

However, under the influence of the English literature, it has been replaced by the term calcar femorale (femoral calcar), used ever since. The term Adams’ arch should be reserved for the thickened medial cortex of the proximal femur, while the term calcar femorale (femoral calcar) should be used for the vertical plate arising from the medial cortex close below the lesser trochanter.

## Introduction

The calcar femorale (femoral calcar) was initially used in the English literature to designate the thickened medial cortex of the femoral neck, receiving primary tension and compression trabeculae from the femoral head [1, 2]. Later, it was also incorrectly used for the medial cortex of the proximal humerus [3]. The desire to denote a clinically important structure by an apt eponym is understandable; nevertheless, the thickened medial cortex of the femoral neck has already been eponymous for more than 175 years, called the *Adams’ arch* [4]. Unfortunately, it has almost been forgotten. Nevertheless, its history is interesting and worthy of restoration.

## Description of the Adams’ arch

**Fig. 1:**
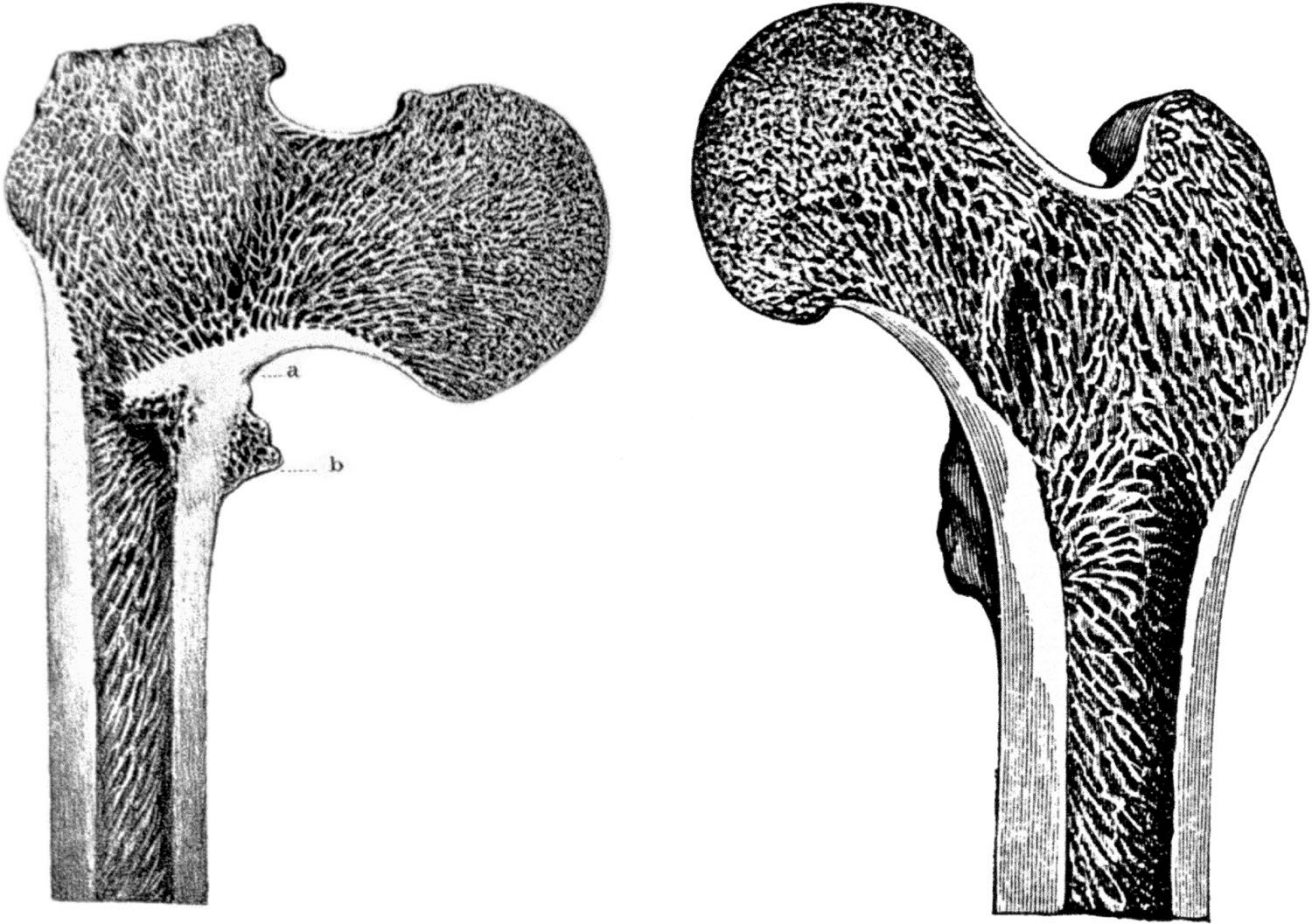
Original drawing of the Adams’ arch published by Smith in 1834

In April 1834, an outstanding Dublin surgeon and anatomist, Robert Adams (1791–1875), presented a lecture on the importance of the thickened medial cortex of the proximal femur in femoral neck fractures. In November of the same year (1834), Robert William Smith (1807–1873), also from Dublin, published a study on femoral neck fractures [5] in which, with Adams’ permission, he included his drawings of this structure (Fig. 1). One year later, in October 1835, the summary of the Adams’ lecture in French was published in Gazette Médicale de Paris [6]. Adams also discussed the importance of this structure in the Todd’s cyclopaedia, published in 1836–1839 [7]. The first to refer to the concept of the two Irish authors was, in 1847, the renowned French surgeon, Joseph-Francois Malgaigne (1806–1865) [8]. He did not agree with Adams in certain aspects of the origin of the so-called incomplete femoral neck fractures, but briefly mentioned his thesis about the importance of the thickened medial cortex of the femoral neck and cited both Adams and Smith. Smith revisited the issue of the thickened medial cortex of the femoral neck in his article of 1840 [9] and, in 1850, in his textbook on fractures [10], although he never used the term “Adams’ arch.”

## Personality of Robert Adams

Robert Adams (1791–1875) was a famous Irish anatomist and surgeon. He cooperated with Abraham Colles (1773–1843) and Robert William Smith (1807–1873) at the Trinity College in Dublin and was member of the Royal College of Surgeons of Ireland [11]. Although they all dealt intensively with fractures of the proximal femur [12], each of them is today remembered in a different context. Both Colles and Smith became famous for their description of fractures of the distal radius [11]. Adams is known for the Adams-Stokes syndrome (syncope triggered by arrhythmia) and primarily for the textbook “Treatise on Rheumatic Gout, or Chronic Rheumatic Arthritis of all the Joints.” However, his contribution to the anatomy of the proximal femur and the treatment of its fractures has fallen into oblivion [13].

## The origin of the term Adams’ arch

**Fig. 2:**
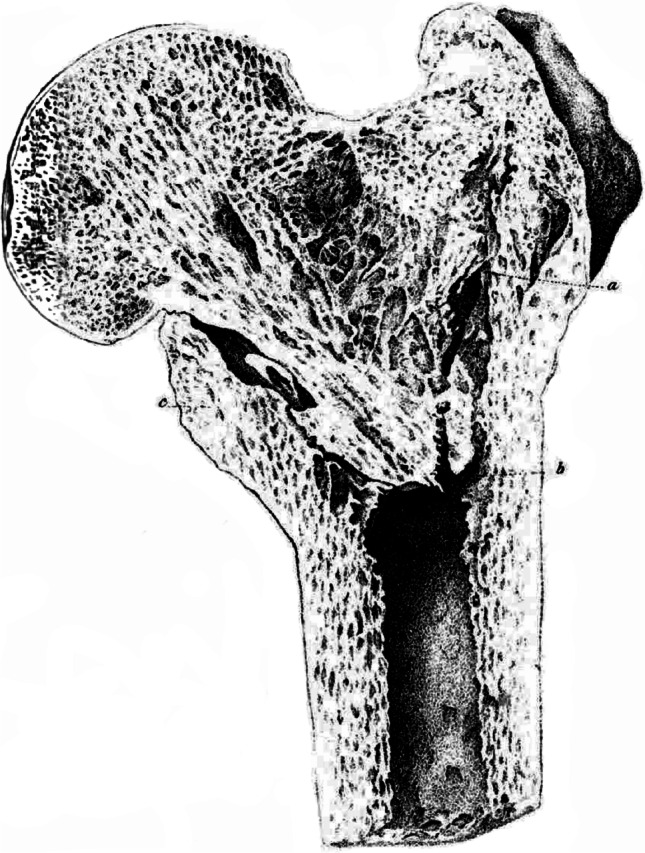
The first drawing of Adams’ arch in the German literature, published by Heppner in 1869

**Fig. 3:**
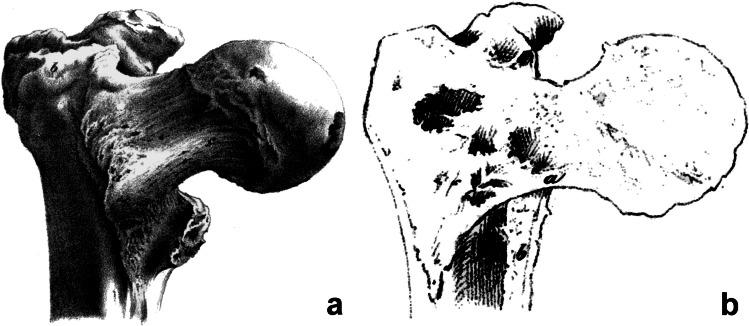
Drawing of the Adams’ arch published by Riedinger in 1874. **a** Anterior aspect; **b** coronal section

The first to use the term Adams’ arch was probably the German surgeon Carl Wilhelm Streubel (1816–1868), in his article focused on experimental femoral neck fractures [4], in which he repeatedly used the terms *Adamscher/Adams’scher/Adam’scher Knochenbogen* and mentioned also Adams and his autopsy findings of proximal femur fractures, without reference, however, to his publications. Another author who applied the term Adams’ arch, or more precisely *Adams’scher Bogen*, in 1869 was **C**. Louis Heppner (?-1874) [14] from St. Petersburg, who mentioned Adams, Smith, and Streubel several times in his article, and also published the first drawing of the Adams’ arch in the German literature (Fig. 2). The same term (*Adams’scher Bogen)* was repeatedly used in 1874 by Ferdinand Riedinger (1844–1919) [15], a surgeon from Würzburg, who referred to the article by Streubel and Heppner (Fig. 3), but did not cite Adams or Smith.

Albert Hoffa (1859–1907), in 1888, used the term *Adam’scher Bogen* without specifying the source, thereby triggering terminological confusion, which has influenced the literature ever since [16]. The Nobel Prize laureate Theodor Kocher (1841–1917) mentioned *Adam’scher Bogen* in his textbook of 1896 [17]. In “Encyklopädie der gesamten Chirurgie” of 1901, Hermann Lossen (1843–1909) from Heidelberg used the term *Adams’scher Bogen* in the chapter on proximal femoral fractures [18] and included the drawing already published by Riedinger. As he obviously knew that article, he used the correct term. Eudard Albert (1841–1900), the Czech surgeon working in Vienna, used alternately the terms *Adam* or *Adams* in his study of the structure of the proximal femur of 1900 [19]. Neither Kocher nor Albert nor Loosen cited their literary sources. Paul Frangeheim (1876–1930), in his outstanding study of 1906 dealing with femoral neck fractures, including trochanteric fractures, repeatedly used the term *Adamscher* or *Adam’scher Bogen* [20].

The cause of mixing up Adams and Adam is documented by Table 1. Only Streubel [4] and Heppner [14] were familiar with the original articles by Adams and Smith. In other authors [17–20], starting Hoffa, based their works on secondary sources and due to a change in the placement of the apostrophe in combination with the German postfix *-schen*, the name of the original author was misspelled. To add to the confusion, in Handlexikon der Medizin [21] of 1980, the term *Adamsbogen* was associated with the prominent English surgeon William Adams (1820–1900).

**Table 1:** Terminology used by authors to designate Adams’ arch

Author	Year	Term	Citation	Language
Streubel	1847	Adamscher/ Adams’scher/ Adam’scher Knochenbogen	Adams	German
Heppner	1869	Adams’scher Bogen	Adams, Smith, Streubel	German
Riedinger	1874	Adams’scher Bogen	Heppner	German
Senn	1883	Adam’s arch Adams’s arch	Heppner	English
Hoffa	1888	Adam’schen Bogen	None	German
Kocher	1896	Adam’schen Bogen	None	German
Albert	1900	Adam Adams	None	Czech
Loosen	1901	Adams’scher Schenkelbogen	None	German
Frangenheim	1906	Adamscher/ Adam’scher Bogen	Heppner, Riendiger	German
Walmsley	1915	Adams’ arch	None	English
Tanton	1916	Arc d’Adams	None	French
Faltin	1924	Adam’s arch	None	English

## USA, Great Britain, France, and Finland

The first non-German speaking author to use the term *Adams’arch* [22] and *Adams’s arch* [23] was the American surgeon Nicholas Senn (1844–1908), who in 1883 also cited exactly the Heppner’s article. In the UK, *Adams’ arch* was mentioned only by the Scottish anatomist Thomas Walmsley (1889–1951) [24] in 1915. One year later (1916), the term *arc d’Adams’* was presented by Jean Tanton (1876–1918) [25]. By contrast, the Finish surgeon Richard Faltin (1867–1952) used the term *Adam’s arch* in his study of proximal femur fractures [26].

## Studies of the structure of the proximal femur in the nineteenth century

**Fig. 4:**
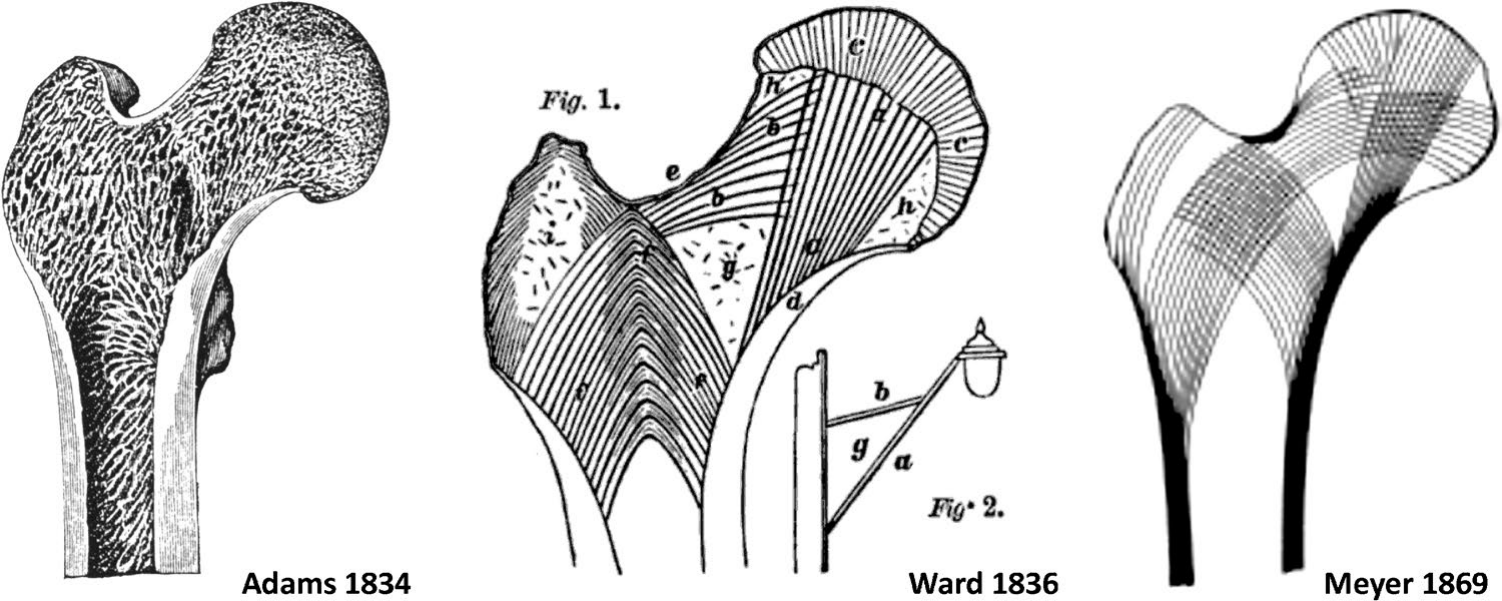
Internal architecture of the femur after Adams [9], Ward [27], and von Meyer [29]

Adams was not the only one at this time to address the structure of the proximal femur. In 1838, a 20-year-old medical student Frederick Oldfield Ward (1818–1877) described in the book *Human Osteology* [27] the structure of the proximal femur accompanied by a drawing (Fig. 4), which has been discussed ever since [28]. Georg Hermann von Meyer (1815–1892), an outstanding German anatomist living in Zurich, published a study “Die Architectur der Spongiosa” in 1867 [29] in which he analyzed in detail the internal architecture of the proximal femur (Fig. 4). The German anatomist Friedrich Julius Wolff (1836–1902) focused, in his monograph “Das Gesetz der Transformation der Knochen” (The law of bone remodeling) of 1892 [30], on the internal architecture of the proximal femur, discussed the Ward’s and von Meyer’s concepts, and summarized all the findings in this field.

However, none of these authors mentioned Adams. The same applies to the major textbook of anatomy of that time [31–35]. It may be explained by the fact that Adams dealt only with the thickened medial cortex of the femoral neck, while Ward and von Meyer discussed the wider structure of the proximal femur in much greater detail.

## Adams’ arch in second half of the twentieth century

Following Faltin’s publication, the eponym Adams’ arch had completely disappeared from the English and French literature.

In the post-World War II German literature, the term *Adambogen* was mentioned by Friedrich Pauwels (1885–1980) in 1965 [36]. In the 1960s, this term became quite common, obviously in connection with development of operative treatment of trochanteric fractures, for example, in Hefte zur Unfalheilkunde N. 106 [37] of 1969, focused on trochanteric fractures. The term *Adambogen* was frequently used by many authors, although their source is not made clear.

The situation was quite different in the AO-literature. The AO founder, **M**aurice Edmond Müller (1918–2009), mentioned *Adambogen* only in his textbook on osteotomies of the hip joint [38]. All AO-Manuals strictly used the term *calcar* [39] or *Kalkar* [40]. An exception was the article by Debrunner and Čech [41], who discussed the importance of *Adambogen* for stability of pertrochanteric fractures. During the 1970s and 1980s, Adambogen gradually disappeared from the German literature, with some exceptions [42], and, under the influence of the English literature, it was replaced by the term *femoral calcar*. In the English literature, only Bombelli [43] used the designation *ADAM’s arch.* Čech and Sosna [44] mentioned Adams’ arch in the classification of subtrochanteric fractures.

## Adams’ arch versus calcar femorale

Sigmund Merkel (1845–1919), in 1874, described a “Schenkelsporn” later called *calcar femorale* [45]. Even if he was not the first to notice this structure [46], he was the first to describe it in detail. Merkel in his description distinguished between the thickened medial cortex of the femoral neck and the vertical bone plate in the region of the lesser trochanter. In the English orthopedic literature, one of the first to deal with the calcar femorale in detail was Kolodny [47] in 1925, who also used the term *internal lamina of the femur* (lamina interna femoris). However, numerous other authors used the term calcar femorale (femoral calcar) to designate the Adams’ arch. Among the first were Titus von Lanz (1897–1967) and Werner Wachsmuth (1900–1990), who in “Praktische Anatomie” of 1938 [48], termed the thickened medial cortex as the calcar femorale and only in the footnote did they include the alternative term *Adamscher Bogen.* Conflating calcar femorale with Adamscher Bogen by these authors is surprising because Merkel’s description was well-known in the German anatomical literature.

**Fig. 5:**
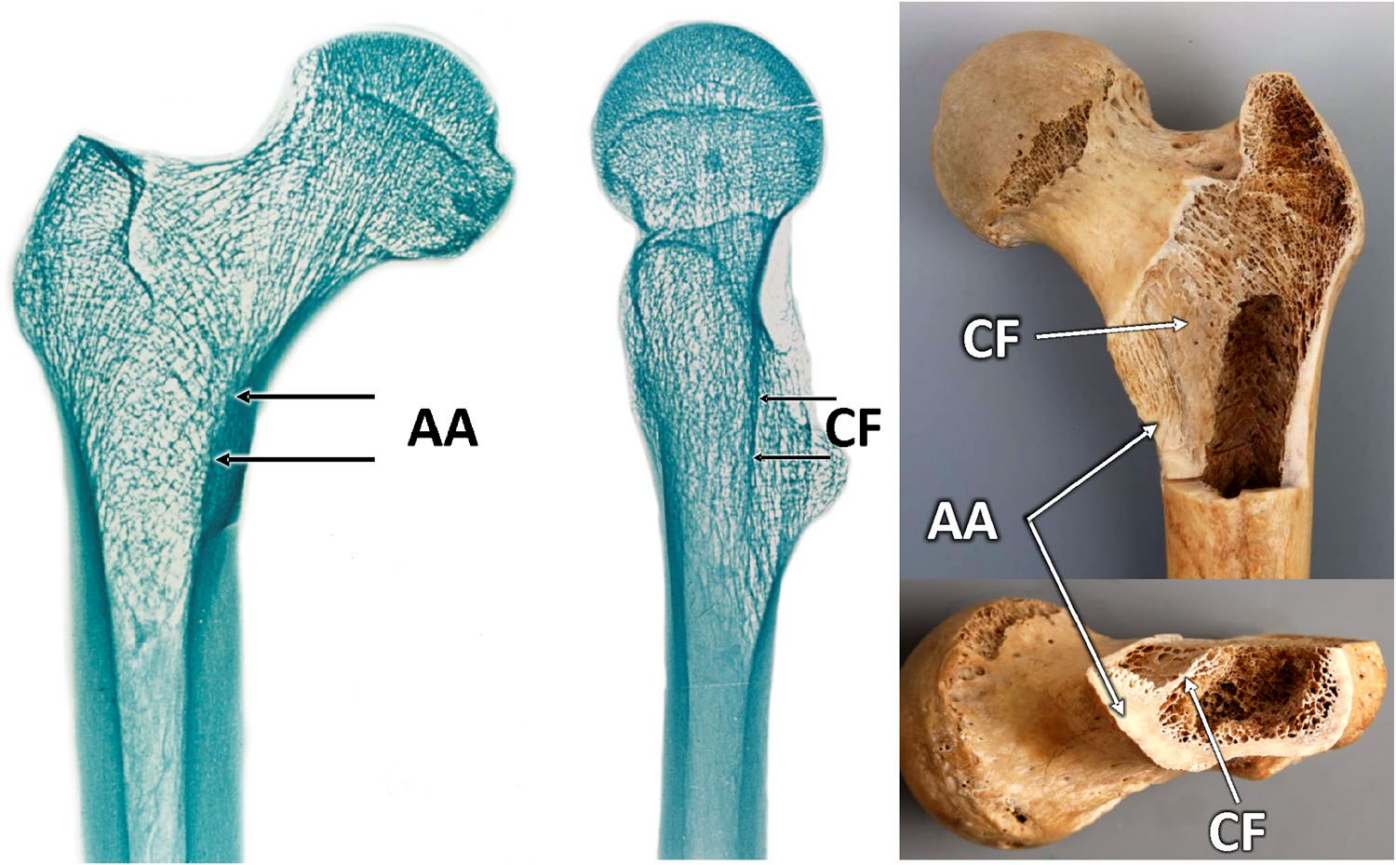
Relationship between the Adams’ arch (AA) and the calcar femorale (CF) on the right proximal femur. The posterior cortex in the region of the lesser trochanter was removed

Evans [49] in his classification of trochanteric fractures of 1949, which later became a model for the AO classification, described the thickened medial cortex of the femoral neck as the calcar femorale. Tobin [50], in his extensive study of the structure of the proximal femur of 1955, described in detail calcar femorale and included its proper drawing, but he considered the adjacent thickened medial cortex to be a part of it. Harty [1], in 1957, followed by Griffin [2] in 1982, pointed out that the calcar femorale and the thickened medial cortex of the femur are two different structures, but to no lasting effect (Fig. 5). “Most orthopaedic surgeons continue to apply the term calcar femorale to the thickened, dense cortical bone of the inferomedial femoral neck at its junction with the shaft, as seen in an anteroposterior radiograph. This is the area of bone particularly concerned with the support of, and transmission of weight from, the femoral component of a total hip replacement” [46].

## Epilogue

There are several reasons why the eponym Adams’ arch failed to take hold or disappeared from the literature. Although the significance of the thickened medial cortex of the femoral neck in extracapsular fractures of the proximal femur was described by the British authors [5, 7], the term *Adams’scher Bogen* appeared for the first time in the German literature and, apart from a few exceptions, did not spread back to the English literature [24]. As it was a clinical term, it did not appear in the anatomical literature, either. It is surprising that Dixon [51], who, similarly to Adams and Smith, worked in Trinity College in Dublin, did not mention Adams’ arch in his study. Garden [52], in 1961, published a thorough anatomical-clinical study on the structure of the proximal femur, including a detailed historical overview of the literature, but without mentioning Adams or Smith.

The clinical need to promote the significance of the thickened medial cortex in trochanteric fractures, and particularly in total hip arthroplasty, with an apt designation has ultimately resulted, despite all warnings, in the use of a wrong term, calcar femorale, which, however, has become established in the clinical literature.

## Data Availability

Not applicable.
